# Potential Implications of the Lung Microbiota in Patients with Chronic Obstruction Pulmonary Disease and Non-Small Cell Lung Cancer

**DOI:** 10.3389/fcimb.2022.937864

**Published:** 2022-07-27

**Authors:** Jia-Qi He, Qin Chen, Sheng-Jun Wu, De-Qin Wang, Shen-Yingjie Zhang, Song-Zhao Zhang, Rui-Lin Chen, Jia-Feng Wang, Zhen Wang, Chen-Huan Yu

**Affiliations:** ^1^ The First Affiliated Hospital of Zhejiang Chinese Medical University, Hangzhou, China; ^2^ Department of Clinical Laboratory Medicine, The Second Affiliated Hospital Zhejiang University School of Medicine, Hangzhou, China; ^3^ Department of Clinical Laboratories, Sir Run Run Shaw Hospital, School of Medicine, Zhejiang University, Hangzhou, China; ^4^ Cancer Hospital of the University of Chinese Academy of Sciences (Zhejiang Cancer Hospital), Hangzhou, China; ^5^ Institute of Basic Medicine and Cancer, Chinese Academy of Sciences, Hangzhou, China

**Keywords:** COPD, NSCLC, lung dysbiosis, complication, clinical trial

## Abstract

Recently, chronic obstructive pulmonary disease (COPD) has been considered as a common risk factor of non-small cell lung cancer (NSCLC). However, very few studies have been conducted on the effects of COPD on the lung microbiota in patients with NSCLC. To identify the lung microbiota in patients with COPD and NSCLC (CN), the microbiome of the induced sputa of 90 patients was analyzed using 16S rDNA sequencing. The results showed no significant differences in the bacterial diversities of induced sputa among patients with COPD, NSCLC, and CN and no intrinsic differences among patients with different pathological types of lung cancer. After surgical operation, the diversities of the induced sputa in patients with CN significantly decreased. More remarkably, both the microbial community phenotypes and the components of the induced sputa in patients with CN obviously differed from those in patients with COPD or NSCLC. The relative abundances of *Streptococcus*, *Veillonella*, *Moraxella*, and *Actinomyces* significantly decreased, but those of *Neisseria* and *Acinetobacter* significantly increased in patients with CN compared with those in patients with COPD or NSCLC alone, resulting in increased Gram-negative microbiota and, therefore, in potential pathogenicity and stress tolerance, as well as in enhancement of microbial glycolipid metabolism, amino acid metabolism, and oxidative stress. Although COPD did not affect the number of pulmonary flora species in patients with NSCLC, these significant alterations in the microbial populations, phenotypes, and functions of induced sputa due to COPD would contribute to inflammation-derived cancer progression in patients with CN.

## Introduction

Chronic obstructive pulmonary disease (COPD) is currently the fourth leading cause of death in the world, and the burden from COPD is expected to increase over the next few decades (Lange et al., 2021). According to the reports of the Pulmonary Health Observational Study in China, there are nearly 100 million COPD patients in the country, which is a heavy burden to the society (Guo et al., 2020; Dong et al., 2021). In addition to irreversible obstructive ventilatory disorders, COPD is also a systemic inflammatory disease, which can also increase the risk of lung cancer. Some studies have shown that the risk of lung cancer in patients with COPD is two to five times higher than that in non-smokers, and the link between the two is not significantly related to age or tobacco exposure (Hou et al., 2019; Parris et al., 2019; Zheng Y. et al., 2021). Moreover, the 5-year overall survival rate of patients with COPD is significantly lower than that of patients without COPD, especially in men and patients with squamous cell carcinoma (Parris et al., 2019; Ahn et al., 2020; Kang et al., 2020). However, a recent study has shown that, compared with never smokers without COPD, the lung cancer incidence rates in never smokers with COPD, ever smokers without COPD, and ever smokers with COPD were increased by 97%, 167%, and 519%, respectively. This indicated that COPD is a strong independent risk factor of lung cancer, irrespective of smoking status (Park et al., 2020).

Growing evidence has demonstrated that the overall changes in pulmonary flora are associated with COPD status (Hou et al., 2019). Through microbiota analysis in pulmonary tissues, bronchoalveolar lavage fluid (BALF), and sputa, the greatest differences in flora between patients with COPD and healthy controls were found to be in *Pseudomonas*, *Streptococcus*, *Prevotella*, and *Haemophilus*, whose relative abundances were significantly elevated with COPD aggravation (Houghton, 2013; Bozinovski et al., 2016; Park et al., 2020). Notably, an increased abundance of TM7 had been found in both COPD and lung cancer patients, indicating that TM7 plays a potential role in the progression of COPD into lung cancer (Cheng et al., 2020; Wang et al., 2021). However, previous reports presented diverse results, and the global perspective of macrobiotic changes from COPD to lung cancer remains to be elucidated.

To date, our understanding of the microbiome in patients with lung cancer is still in its nascent stage. Respiratory samples, such as saliva, sputa, bronchoscopy samples (e.g., bronchial aspirated fluid, BALF, and bronchial mucosa), and lung biopsies, have been widely used in the field of lung microecological research. Given that lung biopsy is invasive and difficult to perform in patients without clinical biopsy indications, induced sputum and BALF samples are easier to implement as noninvasive procedures for dynamically observing the airway microbiome in patients with lung diseases. However, there were obvious differences in the microbiota composition among the above specimens. The microbiota in the upper airway tract differs from that in alveolar tissues, which is partly due to the parenchymal components of the airway and vascular tissues mainly contained in lung tissue samples (Lee et al., 2016; Yang et al., 2021; Zheng Y. et al., 2021; Zitvogel and Kroemer, 2021). The induced sputa obtained from patients with COPD had the most similar compositions to bronchoalveolar aspirated sputa, while BALF had the closest results to the upper bronchial mucosa flora rather than the lower respiratory flora, indicating that induced sputa could be a better representative of lower bronchial dysbiosis in lung diseases (Sze et al., 2012; Huang et al., 2014; Tiew et al., 2021). Therefore, in this study, to investigate the characteristics of the microbiome in patients with COPD and non-small cell lung cancer (NSCLC), 16S ribosomal DNA (rDNA) sequencing was performed to compare the microbiome diversities and differences among the induced sputum samples and to estimate its application value in NSCLC and lung precancerous lesion screening.

## Materials and Methods

### Ethics Approval and Inclusion Criteria

This research was approved by the ethics committees of the participating institution: The First Affiliated Hospital of Zhejiang Chinese Medical University. Informed consent was obtained from all patients. The samples and data were completely anonymized. Basic information was collected on patients’ age, sex, weight, type of pathology, and treatment modality on admission. The study was conducted in accordance with the ethical guidelines and regulations for human research and the Helsinki Declaration.

The inclusion criteria were as follows: 1) lung cancer patients with a clear pathological diagnosis of lung squamous carcinoma or lung adenocarcinoma; benign lung lesions with a clear pathological diagnosis, combined with computed tomography (CT) findings and clinical features; and COPD diagnosis meeting the diagnostic criteria of GOLD 2017 or confirmed diagnosis in the past; 2) age 18–90 years; 3) absence of other types of respiratory infections, such as community-acquired pneumonia, upper respiratory tract infection, acute bronchitis, bronchiectasis with infection, asthma, and acute exacerbation of COPD; and 4) no ongoing antibiotic treatment, immunotherapy, radiotherapy, targeted therapy, or other interventions for tumors.

The exclusion criteria were as follows: 1) patients without clear pathological diagnosis; 2) patients with COPD that could not be clarified through clinical data; 3) patients with lung cancer suspected or clearly combined with lung infection; 4) presence of yellow pus or dark sputum; 5) unknown antibiotic use status prior to specimen collection; 6) patients who received interventions such as immunotherapy, radiotherapy, and targeted therapy prior to specimen collection; and 7) sequencing results that presented insufficient absolute abundance or a homogeneous composition of flora.

After the application of the inclusion and exclusion criteria, a total of 90 patients were eligible for this study, including 67 patients with NSCLC and COPD [18 samples were collected after surgical treatment (CLA group) and 49 samples were obtained from patients not undergoing surgical treatment (CLB group)], 9 patients with NSCLC only (LC), and 14 patients with COPD only. Patients were aged 28–88 years, and the mean age was 64.4 years. There was only one female COPD patient; all other patients in this study were men.

### Collection of BALF Samples

The induced sputum samples from each patient were collected after waking up in the morning. The patients repeatedly gargled three times with normal saline to remove oral bacteria and foreign bodies, and then ultrasonic nebulized 3% saline was inhaled for 15 min. Sputum (2 ml) was expectorated from the deep part of the trachea and collected into sterile Eppendorf tubes. The sputum samples were immediately sent to the laboratory for bacterial smear and sample culture.

Each sputum sample was mixed with two volumes of 0.1% dithiothreitol solution (Merck, Darmstadt, Germany) and then centrifuged at 1,300 rpm for 5 min. Subsequently, two volumes of phosphate-buffered saline (PBS) solution were added to the supernatant and centrifuged again (800 rpm for 5 min). The supernatant was collected and stored at −80°CC. The eligibility criteria for induced sputum were determined as follows: the volume of each sample was >2 ml; the number of epithelial cells was <50% of the total cells; and the number of non-epithelial cells exceeded 200. All of the above processes were performed under sterile conditions.

### DNA Extraction

The sputum was centrifuged at 14,000 rpm for 10 min, the supernatant was discarded, and the pellet was resuspended in 200 μl of sterile PBS and applied to DNA extraction using Blood & Tissue Kit (Qiagen, Hilden, Germany) following the manufacturer’s instructions.

### 16S rDNA Sequencing

The DNA concentration and purity were examined using NanoDrop2000 (Thermo Scientific, Waltham, MA, USA) and the DNA quality tested with 1% agarose gel electrophoresis. Primers 341F (5′-CCTACGGGNGGCWGCAG-3′) and 805R (5′-GACTACHVGGGTATCTAATCC-3′) were used for PCR amplification of the V3–V4 variable regions of 16S rDNA. PCR products were recovered with 2% agarose gel, purified with AMPure XT beads (Beckman, Brea, CA, USA), eluted with Tris–HCl, and detected with 2% agarose electrophoresis. The Quantifluor TM-ST system was used for quantitative detection. Qualified samples were used to construct a PE 2*300 library and sequenced following the standard operating procedures of the Illumina MiSeq Platform.

### Data Analysis

The Trimmomatic software was used for quality control in the original sequencing, splicing was performed with FLASH, and UPARSE (version 7.1) was applied for the operational taxonomic unit (OTU) clustering of the sequences based on 97% similarity. Chimeras were culled with UCHIME. Each sequence was annotated for species classification with the RDP classifier and aligned with the Silva 128/16S bacteria database. The alignment threshold was set to 0.8. The indices *S*_obs_ (observed accumulated richness), Chao, and ACE were adopted to evaluate the richness of the microbial community. The Shannon and Simpson indices were used to assess community diversity. The diversity index was used to comprehensively evaluate the community richness and evenness of the samples. The larger the Shannon value, the greater the community diversity. The smaller the Simpson value, the lower the community diversity. Species composition analysis was performed using I-Sanger.

### Statistical Methods

The paired signed-rank test was used to assess intergroup differences for the α-diversity index and species difference analysis, and statistical analysis was performed using SPSS version 23.0. Principal component analysis (PCA) was applied for the β-diversity analysis to evaluate the similarity and difference between different samples, and statistical analysis was performed using R language. All statistical tests were two-tailed, with *p* < 0.05 considered statistically significant.

## Results

### Analysis of Microbiota Diversity

**Figure 1:**
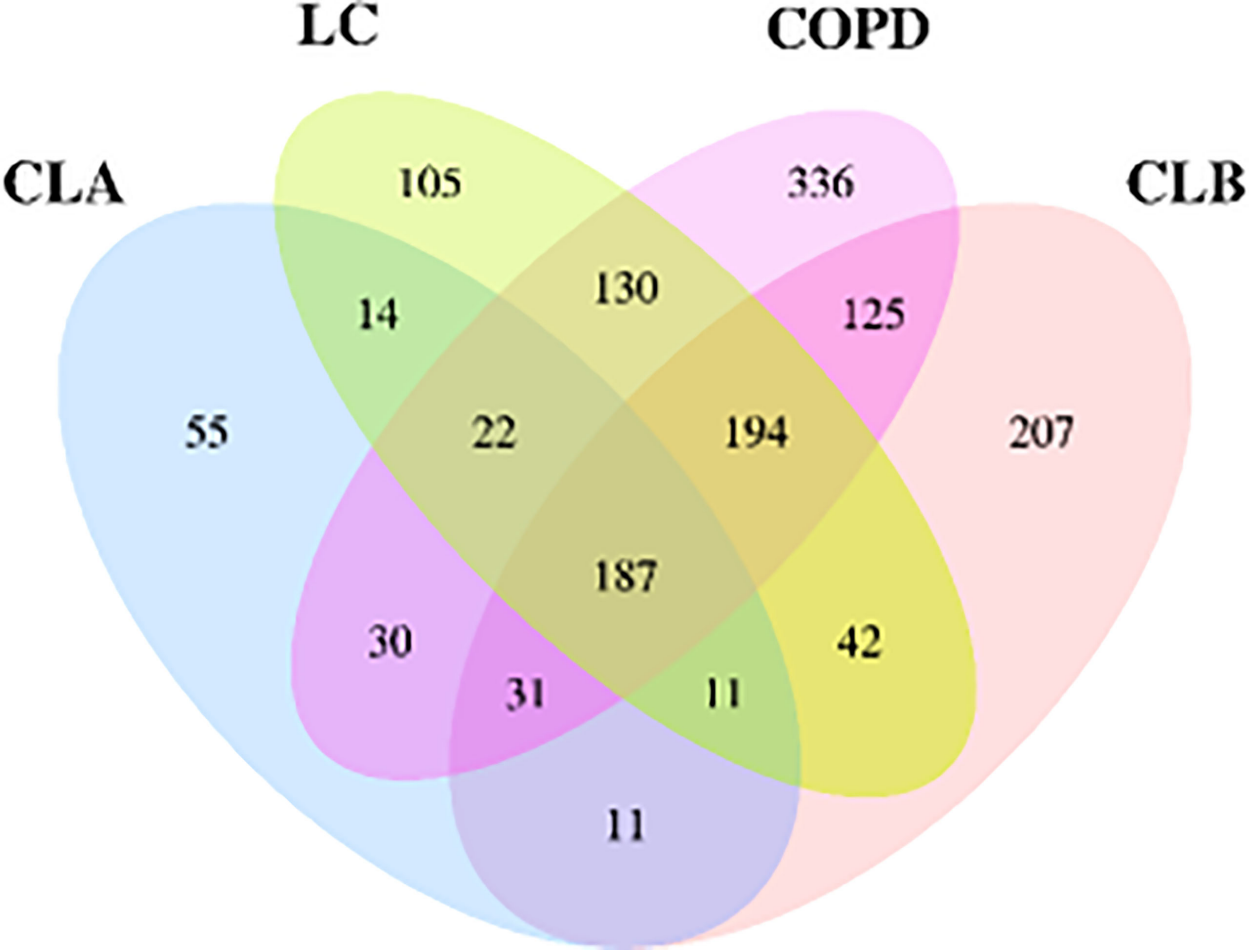
Veen diagram of the number of shared and unique operational taxonomic units (OTUs) among the CLA (surgical treatment), CLB (non-surgical treatment), LC (lung cancer), and COPD (chronic obstructive pulmonary disease) groups.

A total of 1,500 OTUs were obtained using Illumina high-throughput sequencing analysis. To distinguish the shared and unique OTUs in the test groups, a Venn diagram was drawn to visualize the number of OTUs and overlaps between each group. As shown in **Figure 1**, 187 OTUs were shared among the four groups, accounting for 12.5% of the total, whereas 533 OTUs were shared between the lung cancer (LC) and COPD groups and 198 among the LC, CLA, and CLB groups. The OTUs were identified as belonging to 22 phyla and 364 genera.

**Figure 2:**
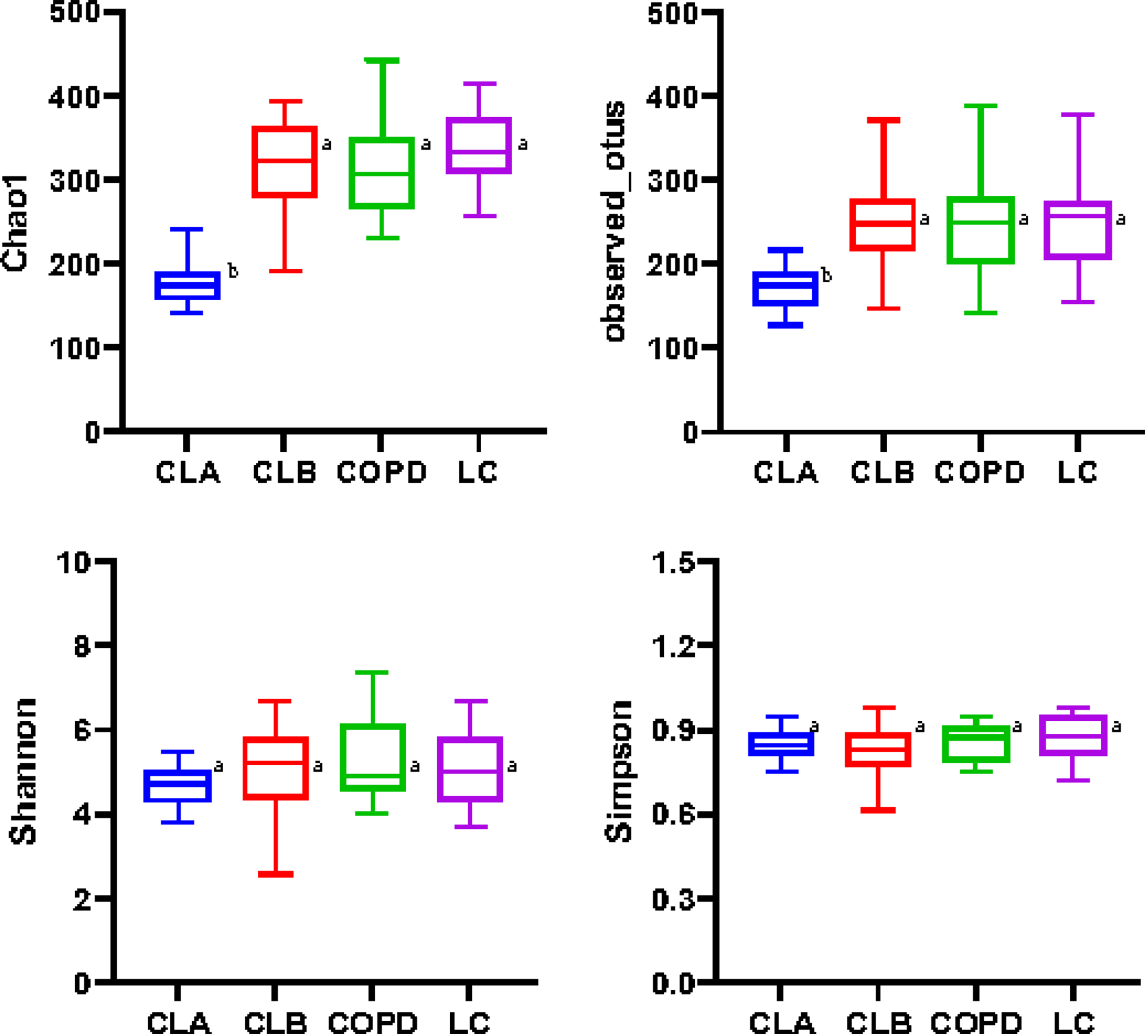
Alpha diversities of the induced sputum samples from different groups. Different indices (Chao1, Observed_otus, Shannon, and Simpson) were used to estimate the number of microbiota in samples. Data shown are the median ± quartile. *Different letters* indicate statistically significant differences. *p* < 0.05.

The Chao1 and Observed_otu indices were positively correlated with the number of species contained in the community, whereas the Simpson and Shannon values indicated the richness and evenness of species, respectively. As shown in **Figure 2**, the values of Chao1 and Observed_otu in the LC group were significantly increased compared with the other three groups (*p* < 0.05). The values in the COPD and CLB groups were very close (*p* > 0.05), but were much higher than those in the CLA group, indicating that the LC group had the highest species diversity, while the CLA group had the lowest. Moreover, there were no significant differences in the Simpson and Shannon values among the four groups, indicating the non-significant difference in the richness and evenness of the lung microbiota among the groups.

**Figure 3:**
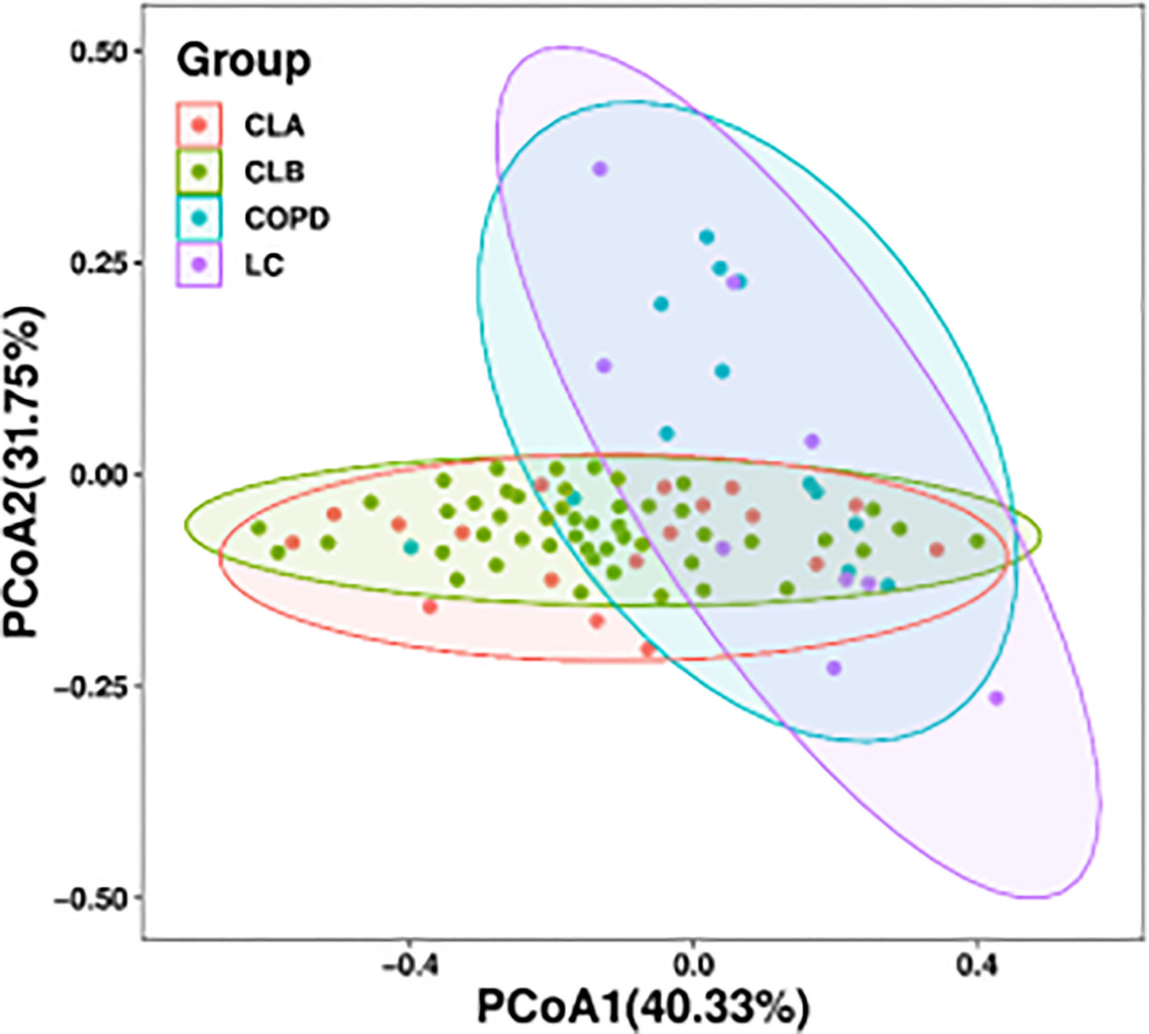
Bacterial diversity clustering by weighted UniFrac principal coordinate analysis (PCoA) of the lung microbiota.

In addition, a principal coordinate analysis (PCoA) graph was obtained by calculating the weighted UniFrac distance (**Figure 3**), which displayed the similarity and difference between the lung microbiome in different environments. Each point in **Figure 2** represents an individual sample, and the distance between points represents the similarity between the samples. In other words, the smaller the distance, the more similar the property. The contributions of the principal components PC1 and PC2 were 40.33% and 31.75%, respectively, based on the weighted principal coordinates of the UniFrac distance, which reflected the overall status of the samples well. Furthermore, samples from the same group presented remarkable discrete states under the same clinicopathological characteristics, indicating that there were individual differences in the microbiome among samples from the same group. Notably, there was a huge overlap between the CLA and CLB groups and between the COPD and LC groups, indicating the similarity of the microbiota composition between the two groups. However, both the COPD and LC groups showed partial overlaps with the combined COPD and LC groups (i.e., CLA and CLB), indicating marked differences among the CLA, CLB, COPD, and LC groups.

### Alterations in the Components of the Lung Microbiome

**Figure 4:**
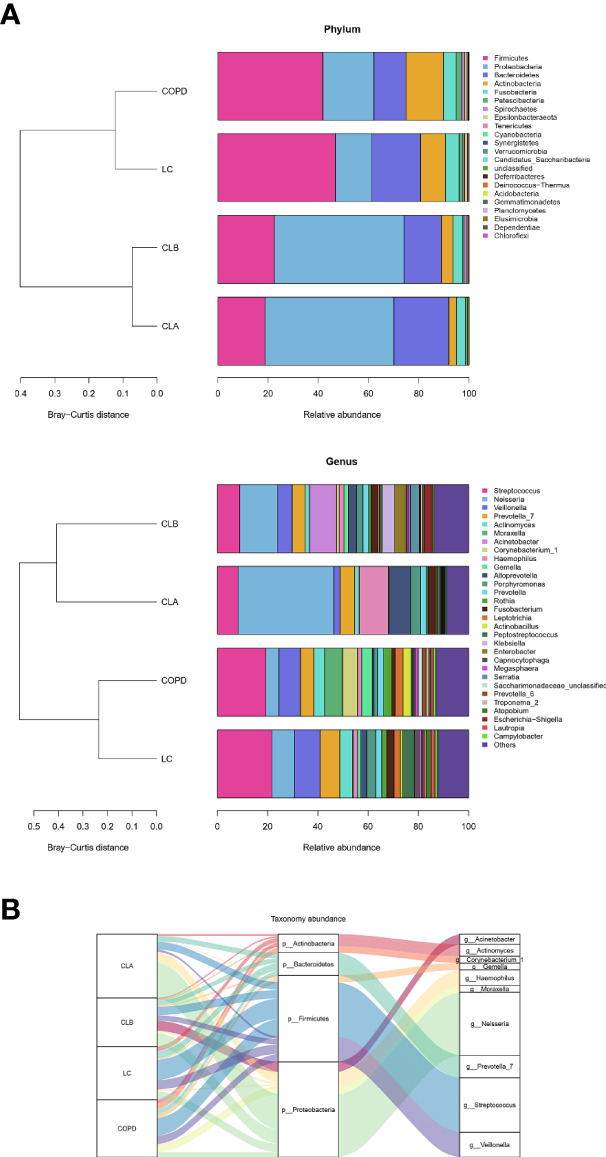
Comparison of the phyla and genera of the microbiomes among the CLA (surgical treatment), CLB (non-surgical treatment), COPD (chronic obstructive pulmonary disease), and lung cancer (LC) groups. **(A)** Comparison of the abundances of the bacterial phyla (*left*) and genera (*right*) of each group. **(B)** Sankey plots of the relative abundances of the discriminatory bacterial phyla and genera among the groups.

**Figure 5:**
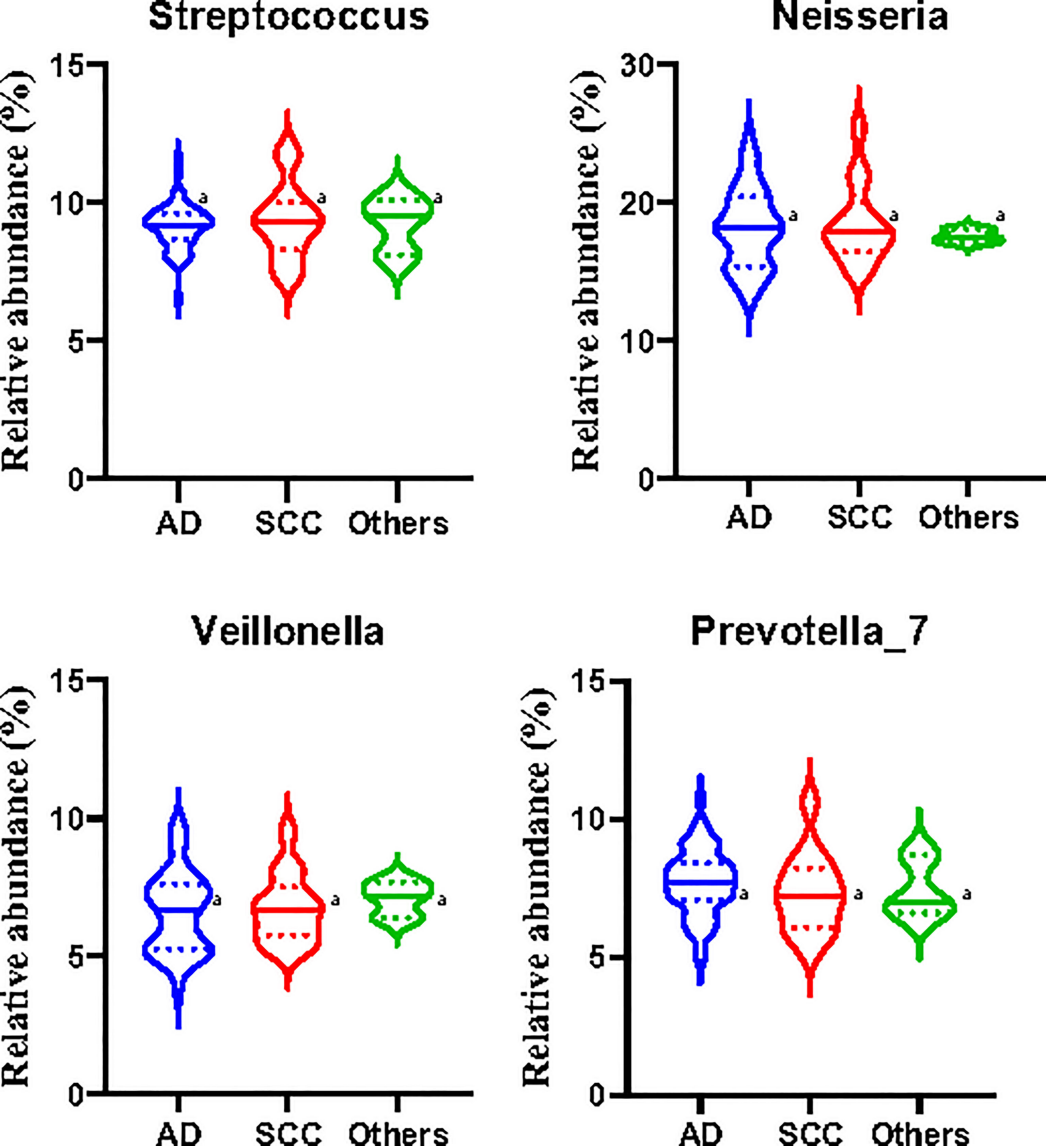
Microbial community phenotypes in the induced sputa of patients with chronic obstructive pulmonary disease (COPD) and/or lung cancer. This community function was predicted using the BugBase web server for quantifying the relative abundance of the microbiome in nine categories. Data shown are the median ± quartile. *Different letters* indicate statistically significant differences. *p* < 0.05.

To visualize the differences in species richness among the groups, we compared the relative abundance of the top 30 species using clustering stacked bar charts (**Figure 4A**). For this purpose, clusters were dynamically generated by merging four groups into two clusters. Interestingly, at both the genus and phylum levels of taxonomic criteria, CLA and CLB were grouped into one cluster, whereas COPD and LC comprise another cluster. These results demonstrate that the relative abundances of the top 30 microbial compositions at the genus and phylum levels were not significantly altered by surgical operation. More importantly, the microbial compositions of the COPD and NSCLC combination groups differed from those of the COPD and LC groups, indicating the high specificity of COPD patients with lung cancer. The dominant phyla were Firmicutes, Proteobacteria, Bacteroidetes, Actinobacteria, and Fusobacteria; especially, the levels of the first two accounted for >60% of the total. On the other hand, the dominant genera in these four groups were *Streptococcus*, *Neisseria*, *Veillonella*, *Prevotella_7*, *Actinomyces*, *Moraxella*, *Acinetobacter*, *Corynebacterium_1*, *Haemophilus*, *Gemella*, *Alloprevotella*, *Porphyromonas*, *Rothia*, *Fusobacterium*, and *Leptotrichia*, all of which accounted for more than nearly 70% of the total abundances. Remarkably, the relative abundances of *Veillonella*, *Haemophilus*, *Alloprevotella*, and *Acinetobacter* in the CLA group were significantly elevated compared with the other three groups, indicating obvious alterations in the Gram-negative microbial compositions, which was found to be in agreement with the results of BugBase (**Figure 5**).

To further display the specific flora visually in the different groups of patients, a Sankey diagram was plotted to demonstrate the taxonomic abundance of the main lung microbiota. As shown in **Figure 4B**, *Streptococcus*, *Neisseria*, *Veillonella*, and *Prevotella_7* were the main species in the induced sputa of patients. Compared with the other groups, the relative abundances of *Streptococcus* and *Actinomyces* were significantly elevated in the LC group. *Moraxella*, *Corynebacterium_1*, and *Gemella* had high expressions in the COPD group, whereas these had little or no expression in the LC, CLA, and CLB groups, whose data were consistent with those of previous reports (Leung et al., 2017; Beech et al., 2020). Compared with the other groups, *Haemophilus* and *Neisseria* showed the highest levels in the CLA group, whereas *Acinetobacter* was only observed in the CLB group. These results revealed that induced sputum from different types of patients had different microbiota profiles, which could be used as special diagnostic markers and therapeutic targets for the prevention of COPD and lung cancer.

**Figure 6:**
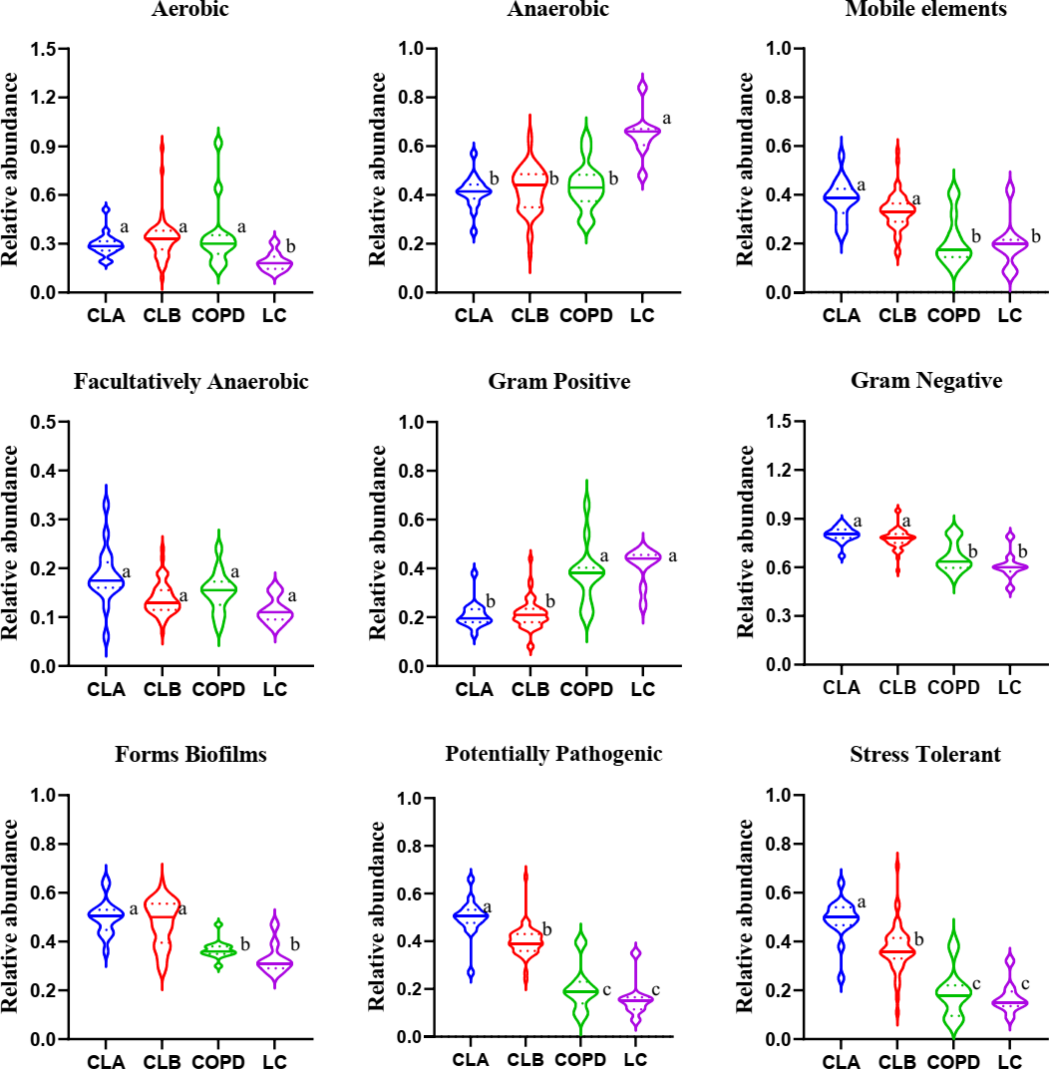
Relative abundances of the main microbiota in the induced sputa of patients with non-small cell lung cancer (NSCLC). *AD*, patients with lung adenocarcinoma; *SCC*, patients with squamous cell carcinoma; *Other*, except AD and SCC, patients with other subtypes of NSCLC. Data shown are the median ± quartile. *Different letters* indicate statistically significant differences. *p* < 0.05.

However, there were no significant differences in the relative abundances of *Streptococcus*, *Neisseria*, *Veillonella*, and *Prevotella_7* among patients with lung adenocarcinoma, squamous cell carcinoma, and other subtypes of NSCLC (**Figure 6**). The limited number of samples collected in the study may have affected the accuracy and representation of the outcome, and further studies need to be developed in the future.

### Alterations in the Function of the Lung Microbiome

**Figure 7:**
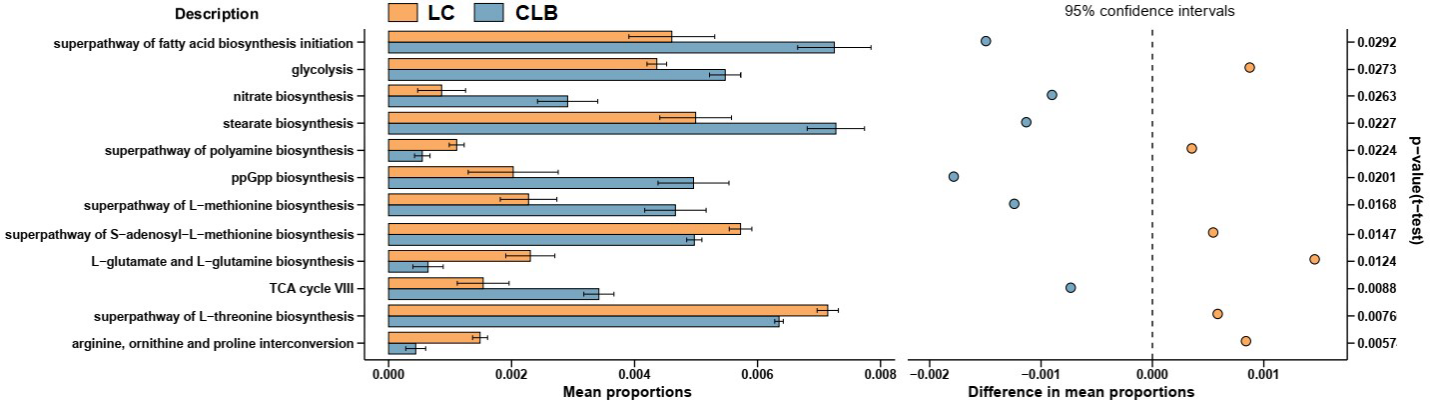
Function alterations of the lung microbiota between patients with lung cancer only (LC) and patients with both COPD and lung cancer (CLB). Microbial gene functions were predicted by using the PICRUSt2 algorithm.

As shown in **Figure 7**, the functions of the differently expressed lung microbiome were analyzed using the PICRUSt2 algorithm, and their biological annotations were referenced from the KEGG database. Compared with those of the LC group, the rates of l-glutamate and l-glutamine biosynthesis, superpathway of l-threonine biosynthesis, superpathway of polyamine biosynthesis, superpathway of *S*-adenosyl-l-methionine biosynthesis, and of arginine, ornithine, and proline interconversion significantly decreased in the CLB group (*p* < 0.05), but the rates of superpathway of fatty acid biosynthesis initiation, superpathway of l-methionine biosynthesis, TCA cycle, glycolysis, stearate biosynthesis, ppGpp biosynthesis, and nitrate biosynthesis significantly increased in the CLB group (*p* < 0.05). This indicates that the abnormal alterations in glycolipid metabolism, amino acid metabolism, and oxidative stress, which are mediated by lung microbes, would contribute to inflammation-driven lung cancer.

## Discussion

On the surface, COPD and lung cancer are two distinct diseases. COPD is characterized by chronic lung injury with two main manifestations—airflow limitation and parenchymal destruction—which are often accompanied by increased apoptosis, autophagy, and senescence caused by smoking. In contrast, lung cancer is characterized by abnormal DNA damage and genomic instability, leading to tumor angiogenesis and immune escape. However, a lot of studies now suggest that the pathogenesis between the two diseases has some commonalities, tobacco smoke inhalation being the most common trigger (Szalontai et al., 2021). In addition, immune dysfunction, lung microbiota dysbiosis and inflammatory infections, oxidative stress, and DNA damage play a role in the development of COPD and lung cancer (Caramori et al., 2019; Hou et al., 2019; Parris et al., 2019; Sears, 2019), all of which may be potential drivers of the progression of COPD into lung cancer.

Clinical and animal studies have revealed tumor-associated dysregulation of the local microbiome in the lung, which in turn impacted cancer progression through systemic inflammatory response (Weinberg et al., 2020; Zitvogel and Kroemer, 2021). In addition, epidemiological evidence revealed that the repeated use of antibiotics would induce an increased risk of lung cancer, suggesting that abnormal pulmonary microbial communities could play a part in the occurrence of lung cancer. However, the association between pulmonary flora and lung cancer remains unclear. As yet, systematic and large-scale clinical observation of lung dysbiosis in patients with COPD and NSCLC has been lacking. In this study, the microbial α-diversity in the induced sputa of CLB patients was not significantly higher than those in patients with COPD and NSCLC (*p* > 0.05), but the diversity in CLA patients was obviously decreased compared with that of the other three groups, indicating that the microbial species were reduced after surgical operation. Furthermore, *Streptococcus*, *Neisseria*, *Veillonella*, *Prevotella_7*, *Actinomyces*, *Moraxella*, *Acinetobacter*, *Haemophilus*, and *Gemella* were the dominant bacteria in the induced sputa of all these patients, whose populations varied with the disease type. Compared with those in patients with COPD and LC, the relative abundances of *Streptococcus*, *Veillonella*, *Actinomyces*, *Corynebacterium_1*, *Rothia*, and *Leptotrichia* significantly decreased in the CLB group, whereas the abundances of *Neisseria*, *Haemophilus*, and *Alloprevotella* significantly increased. After tumor resection, the levels of *Haemophilus* and *Neisseria* were drastically elevated in the CLA group. Particularly, a high level of *Acinetobacter* was only observed in the CLB group, indicating its specificity in the progression of COPD-related lung cancer.

The lung microbiome regulates inflammatory factors in lung tissue by producing oncogenic metabolites and toxins, which can bind to Toll-like receptors on antigen-presenting cells such as monocytes and dendritic cells, inducing chronic inflammation, which in turn disrupts the cell cycle, leading to the upregulation of oncogene signaling pathways and promoting lung carcinogenesis (Nowrin et al., 2014; Mendez et al., 2019; Weinberg et al., 2020; Zitvogel and Kroemer, 2021). Through analysis with the BugBase and PICRUSt2 algorithms, the phenotypes and the functions of the mainly differentially expressed microbiota among patients with different pathological types of lung diseases were predicted, respectively. The results demonstrated that the Gram-negative microbiota, potential pathogenicity, and the stress tolerance of the microbiota in the COPD-combined NSCLC patients significantly increased compared to those in patients with COPD or NSCLC only. Moreover, the microbial glycolipid metabolism, amino acid metabolism, and oxidative stress of such perturbed lung microbial community were also enhanced. During inflammation and cancer, host immune cells or tumor cells need energy to support their physiological function, leading to increased anaerobic glycolysis and amino acid consumption (Fahrmann et al., 2020; Schiliro and Firestein, 2021; Stepka et al., 2021; Tomé, 2021), which was consistent with our findings on the metabolic alteration of the lung microbiome. Thus, these changes may be associated with specific metabolic characteristics of COPD-related lung cancer.

Unlike in Western countries, most smokers in China are men (Yang et al., 2020). Thus, it was difficult to collect samples from the same number of male and female patients, resulting in an imbalance in this study. Similarly, the chances of developing cancer increase with age (Toumazis et al., 2020). Most patients with NSCLC in this study were retired. Recently, sex and age differences have been demonstrated to exert a direct influence on oncological treatments, specifically immunotherapy, with documented distinctions between men and women (Vavalà et al., 2021). Consequently, to correctly assess cancer outcomes, a multicenter study with greater population is required in the future.

In conclusion, the microbial populations, phenotypes, and functions of induced sputa exhibited intrinsic differences among patients with COPD, NSCLC, and CN. Compared with patients with COPD and NSCLC alone, the relative abundances of *Streptococcus*, *Veillonella*, *Moraxella*, and *Actinomyces* in patients with CN were significantly reduced, but those of *Neisseria* and *Acinetobacter* were significantly elevated, resulting in increased potential microbial pathogenicity and energy metabolism. The results showed that COPD may affect the populations of pulmonary microbiota in patients with lung cancer, and drastic alterations in the phenotypes and functions of induced sputa among the different pathological types of lung cancer were also related to the presence of COPD.

## Data Availability Statement

The datasets analyzed for this study can be found in the repository Jinguoyun https://www.jianguoyun.com/p/Dft7UUEQ5e3kChiNzs4EIAA.

## Ethics Statement

The studies involving human participants were reviewed and approved by the First Affiliated Hospital of Zhejiang Chinese Medical University. The patients/participants provided written informed consent to participate in this study.

## Author Contributions

J-QH, QC, ZW, and C-HY conceived and directed the study. J-QH, QC, S-ZZ, S-YZ, and J-FW did the analysis and visualization. S-JW, QC, D-QW prepared the sample, and R-LC prepared the samples. J-QH and C-HY drafted the manuscript. ZW and CH-Y revised the manuscript. All authors agree to be accountable for the content of the work. All authors contributed to the article and approved the submitted version.

## Funding

This work is supported by Medical Science and Technology Project of Zhejiang Province (No. 2022KY917).

## Conflict of Interest

The authors declare that the research was conducted in the absence of any commercial or financial relationships that could be construed as a potential conflict of interest.

## Publisher’s Note

All claims expressed in this article are solely those of the authors and do not necessarily represent those of their affiliated organizations, or those of the publisher, the editors and the reviewers. Any product that may be evaluated in this article, or claim that may be made by its manufacturer, is not guaranteed or endorsed by the publisher.
